# Recognition of Heavy Metals by Using Resorcin[4]arenes Soluble in Water

**DOI:** 10.3390/toxics10080461

**Published:** 2022-08-09

**Authors:** Edilma Sanabria, Miguel A. Esteso, Edgar F. Vargas

**Affiliations:** 1 Grupo GICRIM, Programa de Investigación Criminal, Universidad Manuela Beltrán, Avenida Circunvalar No. 60-00, Bogota 111321, Colombia; 2 Universidad Católica de Ávila, Calle los Canteros s/n, 05005 Ávila, Spain; 3 Unidad Docente Química Física, Universidad de Alcalá, 28805 Alcalá de Henares (Madrid), Spain; 4 Departamento de Química, Universidad de los Andes, Cr. 1 No. 18A 10, Bogota 111711, Colombia

**Keywords:** heavy metals, inorganic pollutants, resorcin[4]arenes, recognition

## Abstract

The complexing properties of two water-soluble resorcin[4]arenes (tetrasodium 5,11,17,23-tetrakissulfonatemethylen 2,8,14,20-tetra(butyl)resorcin[4]arene, Na_4_BRA, and tetrasodium 5,11,17,23-tetrakissulfonatemethylen-2,8,14,20-tetra(2-(methylthio)ethyl)resorcin[4]arene, Na_4_SRA) with polluting heavy metals such as Cu^2+^, Pb^2+^, Cd^2+^ and Hg^2+^ were studied by conductivity, and the findings were confirmed by using other techniques to try to apply this knowledge to removing them. The results indicate that Na_4_BRA is able to complex Cu^2+^ in a 1:1 ratio and Pb^2+^ in a 1:2 ratio, while Na_4_SRA complexes Hg^2+^ in a 1:1 ratio. On the contrary, no indications have been observed that either of the resorcin[4]arenes studied complexes the Cd^2+^ ions. The results suggest that the bonds established between the sulfur atoms located at the lower edge of the SRA^4−^ and the solvent hydrogens could prevent the entry of the guest into the host cavity. However, in the case of Hg^2+^ ions, the entry is favoured by the interactions between the sulfur donor atoms present on the lower edge of Na_4_SRA and the Hg^2+^ ions. Therefore, it can be said that Na_4_BRA is selective for Cu^2+^ and Pb^2+^ ions and Na_4_SRA is selective for Hg^2+^ ions.

## 1. Introduction

Soil contamination by heavy metals poses a great risk to human health, animals, plants, microorganisms and their interactions [1]. In humans, for example, prolonged exposure to contaminated soils has negative effects on the central nervous system, the gastric system and the respiratory system [2,3]. Additionally, heavy metals can cause great ecological risk when they are absorbed by different aquatic organisms, thus entering the food chain [4]. They also affect the functioning of soil enzymes and microbial biomass (affecting microorganisms is crucial since they play an important role in the cycle of nutrients and in the decomposition of matter), hindering their growth and consequently degrading the quality of the soil. Examples of the above are those produced by Pb, which has effects on bacteria, and by Cd, which impacts the fungal population [5]. The fate and effect of these pollutants depend on the geographical and environmental conditions, the nature of the soil and the type of human activities, including the use of agrochemicals [6,7], mining [8,9,10,11] and industrial activities [12,13].

Aware of the problem, this research seeks to show progress in the recognition of certain polluting heavy metals in solution, such as Cu^2+^, Pb^2+^, Cd^2+^ and Hg^2+^, using complexing macrocycles such as the resorcin[4]arenes, to try to apply it in their removal. Although, when Cu is present in the human body in amounts that do not exceed 75–100 mg, it is vital for health, when the concentration of this metal is higher, various disorders occur such as nausea, vomiting, abdominal pain and cramps, headache, dizziness, weakness and diarrhea, among others; such disorders occur mainly in the case of individuals with liver diseases and other pathologies in which the excretion of bile is compromised [14]. The effect of lead (Pb) on humans is also important, since it acts on the calcium and potassium channels in the cell membrane, affecting coordinated cellular functioning and giving rise to neuropsychological disorders [13,15]. Likewise, cadmium (Cd) is classified within the first group of carcinogens by the International Agency for Research on Cancer (IARC) as a highly toxic metal [16,17]. It also causes decreased bone density and kidney damage [18]. Finally, mercury is a toxic metallic element that affects human health, wildlife, aquatic ecosystems (severe and persistent toxicity has been documented from the contaminated fish consumption from fresh and marine waters [4]) and, in general, soils, water and air [19,20,21,22,23,24].

**Figure 1:**
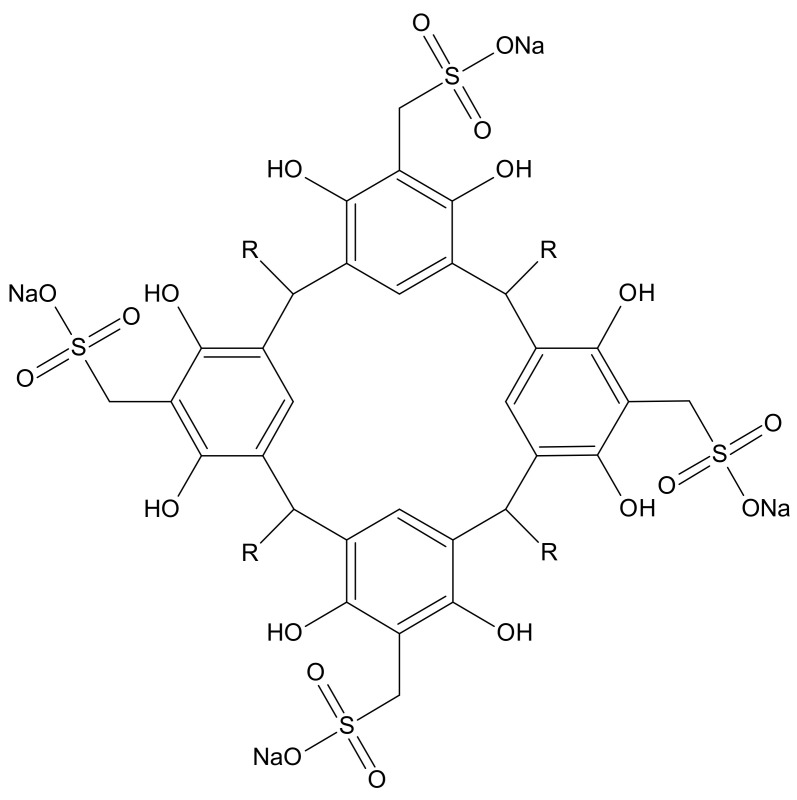
Resorcin[4]arene sulfonate structure (Na_4_RA).

**Figure 2:**
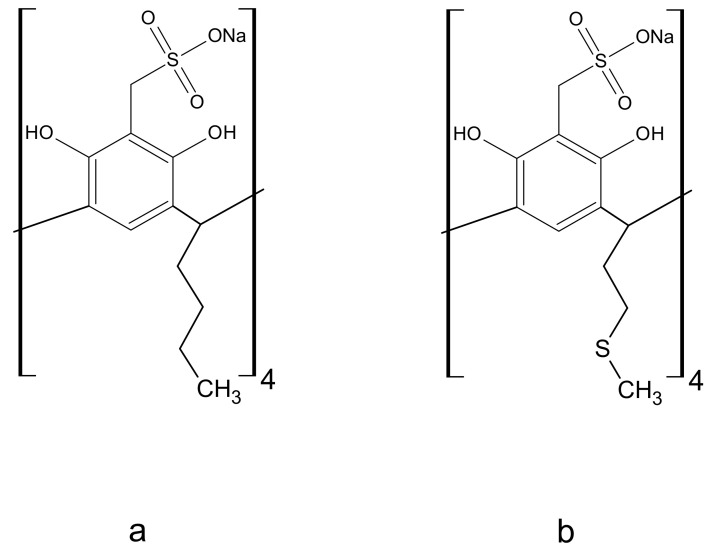
Resorcin[4]arenes sulfonated, whose complexing properties with Cu^2+^, Pb^2+^, Cd^2+^ and Hg^2+^ were evaluated. (**a**) Tetrasodium 5,11,17,23-tetrakissulfonate methylen-2,8,14,20-tetra(butyl)resorcin[4]arene (Na_4_BRA). (**b**) Tetrasodium 5,11,17,23-tetrakissulfonatemethylen-2,8,14,20-tetra(2-(methylthio) ethyl)resorcin[4]arene (Na_4_SRA).

Resorcin[4]arenes (Figure 1) and their functionalized derivatives have been widely used for complexation studies. Both the selectivity of these ligands towards a given guest [25,26,27,28] and the stability of the complexes formed are determined by factors such as geometric complementarity, the types of interactions [25,29] and the nature of the substituents in the macrocycle [30,31,32,33]. The stability of the complex is determined by the magnitude of the complex formation constant, *β*_ƒ_ [34,35]. Information about host-guest interactions can be derived from its value. The macrocycles evaluated were tetrasodium 5,11,17,23-tetrakissulfonatemethylen 2,8,14,20-tetra(butyl)resorcin[4]arene (Na_4_BRA) and tetrasodium 5,11,17,23-tetrakissulfonatemethylen-2,8,14,20-tetra(2-(methylthio)ethyl) resorcin[4]arene (Na_4_SRA), whose structures are shown in Figure 2.

There are several simple experimental techniques that can be used to determine the stoichiometry and stability constants of complex species. Among the most widely used are conductometry, sonometry, ion-selective potentiometry (ISE), acid-base potentiometry, nuclear magnetic resonance (NMR), UV-VIS spectroscopy and isothermal titration calorimetry (ITC). In the present case, the techniques used to study the complexation of Na_4_BRA and Na_4_SRA with the heavy metals of interest are shown in Table 1.

The process of recognition and evaluation of the selectivity of the resorcin[4]arenes was carried out in water by using the conductivity technique, and the positive findings were confirmed by other techniques such as ion-selective potentiometry (ISE) and atomic absorption. The results are discussed in terms of the structure and three-dimensional arrangement of each macrocycle.

## 2. Materials and Methods

### 2.1. Materials

Na_4_BRA and Na_4_SRA were synthesized, purified and characterized according to the procedures described by Sanabria et al. [36]. Heavy metal perchlorates used as guests were used as supplied without further purification; they were stored in amber flasks and dried *in vacuo* over activated silica gel; their purities are shown in Table 2. Water used to prepare the solutions for the conductivity complexation, potentiometry and atomic absorption tests was obtained from a Milli-Q purifier, and it was degassed before use; its conductivity was always <0.1 μS·cm^−1^. The solutions were prepared by weight and corrected to vacuo using an OHAUS Analytical Plus balance (AP250D) (Ohaus Corporation, Florham Park, NJ, USA) that has an accuracy of 1 × 10^−5^ g in the range of 80 g and 1 × 10^−4^ g in the range from 80 g up to 250 g.

### 2.2. Equipments and Experimental Techniques

#### 2.2.1. Conductometry

Conductometry has been commonly used in complexation studies between macrocycle ligands and different types of ions [37,38]. The stability constants found by conductometry have been reported in the literature for various macrocycles, among which are: α and *β*-cyclodextrins [39,40], alkylcalix[4]arenes [41] and crown ethers [41,42].

Conductivity measurements were carried out using a VEB hydromat Bannewitz LM 3000 cell (Bannewitz, Germany) with a cell constant of K_cell_ = 1.04 cm^−1^. It is made of borosilicate glass and provided with two platinum electrodes. The cell was placed inside a 120 mL jacketed glass vessel and kept at a constant temperature of (298.15 ± 0.01) K, by circulating water through the outer jacket, using a Julabo LC6 temperature controller (Julabo Labortechnick, Seelbach, Germany). The cell was carefully washed, purged with deionized water and dried at 105 °C before use. To avoid the presence of CO_2_, nitrogen gas was passed through the cell before performing each determination. Resistance measurements were carried out with a Stanford SR720 (US) LCR meter (Stanford Research Systems, Sunnyvale, CA, USA) whose accuracy is 0.05%. A voltage of 1.0 V, at a frequency of 1 kHz, was used for all measurements. In a typical complex formation experiment, 40 mL of approximately 1 × 10^−3^ M Na_4_RA aqueous solution were placed into a titration cell and thermostated at 298.15 K. Once the solution reached thermal equilibrium, the resistance of the solution was measured with the LCR meter, and then it was titrated with a ten-times more concentrated guest solution (approximately 1 × 10^−2^ M). This titration solution was added in 0.1 mL portions, using a metrohm (Metrohm Ltd., Herisau, Switzerland) digital burette, until a ratio of 5/1 (guest/host) was reached. After each addition, the solution was stirred for 20 s, allowed to stand for another 20 s, and its resistance was recorded. The above procedure was carried out with all the proposed metals. A blank was run for each experiment using water instead of the Na_4_RA solution [25].

#### 2.2.2. Ion-Selective Potentiometry (ISE)

Ion-selective electrodes have been successfully used for the determination of complex stability constants [43]. The general procedure consists of carrying out titration, at a constant ionic strength of a solution of the guest with the host, while measuring the potential. The formation of 1:1 complexes of Na_4_RA with a M^2+^ cation can be represented by the equilibrium:

H_8_L^4−^ + M^2+^ ↔ H_8_LM^2−^ (1)

whose stability constant, at pH 7, can be obtained using the hyperquad software (Wiley Organics, Inc., Coshocton, OH, USA) [44].

Potentiometric titration was carried out only with the Na_4_BRA-Cu^2+^ system. For this, a Cu^2+^ ion-selective electrode (Cu-ISE, Orion model 94-29, (ThermoFisher Scientific, Waltham, MA, USA) filled with 0.1 N potassium nitrate solution) was used. As in the case of conductivity titrations, it was carried out in a 120 mL jacketed glass cell, and the temperature was kept constant at 298.15 K by circulating water, through the said jacket, from a bath equipped with a Julabo LC6 thermostat (Julabo Labortechnick, Seelbach, Germany), that ensures a temperature control of ±0.001 degrees. The calibration curve was prepared with solutions of 0, 1, 10, 100, 300, 600, 800 and 1000 ppm and the ionic strength was controlled with 0.01M NaClO_4_. For the sample analysis, 20 mL of 2.9 × 10^−4^ M Cu(ClO_4_)_2_ solution were placed inside the titration vessel thermostated at 298.15 K; once equilibrium was reached, the sample was titrated using a Metrohm burette (Metrohm Ltd., Herisau, Switzerland) with an 8.3 × 10^−4^ M Na_4_BRA solution [25]. The sample voltage was recorded after each addition, and subsequently, the value obtained was interpolated in the calibration curve to determine the Cu^2+^ concentration.

#### 2.2.3. Atomic Absorption

This is not a technique used to find the formation constant of a complex, but it allows to determine in a simple, fast and selective way the percentage of metal present in a complex and thus verify the stoichiometric ratio determined by other techniques.

The conductometric titration of both Na_4_BRA with Pb(ClO_4_)_2_ and Na_4_SRA with Hg(ClO_4_)_2_ produced precipitates three hours after the end of the titration. In both cases, the precipitate was filtered, washed with plenty of water, dried and analyzed by atomic absorption. For this analysis, a Perkin Elmer Analyst 300 spectrophotometer (Perkin Elmer Ltd., Wembley, UK) with an air-acetylene flame in a 10:2 ratio, 0.70 slot, and AAwinlab software was used. The corresponding calibration curve for each metal ion was created by dilution from a concentrated solution of 1000 ppm of Me(NO_3_)_2_; the final concentrations obtained were: 1, 4, 8, 12, 16 and 20 ppm of Me^2+^. The complex with either Pb or Hg was digested with nitric acid and afterwards analyzed. The absorbance of the sample was determined in triplicate and the concentration was obtained by interpolation in the corresponding calibration curve [25].

## 3. Results and Discussion

The complexation measurements were carried out in water at 298.15 K and pH = 7. According to the species distribution diagram, under these conditions, the predominant species is H_8_L^4−^ for both Na_4_BRA and Na_4_SRA [25].

### 3.1. Complexation of Na_4_BRA with Cu^2+^

**Figure 3:**
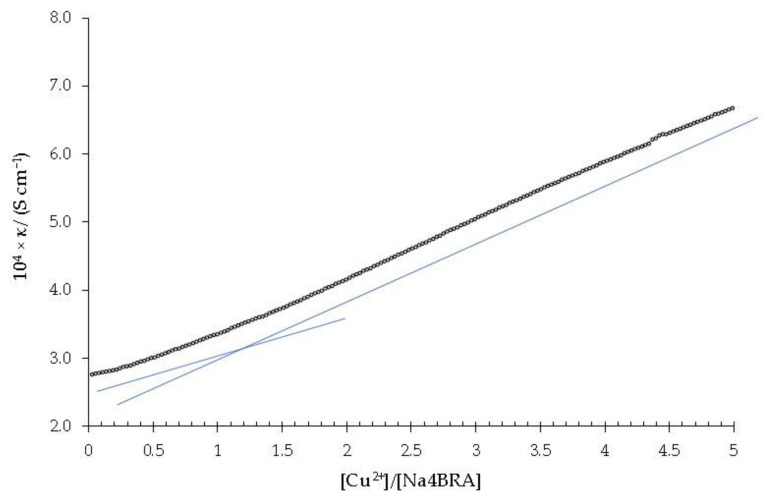
Specific conductivity *versus* the [Cu^2+^]/[Na_4_BRA] ratio. The solid blue lines (which are represented displaced parallel for better visualization) are shown as a visual aid to indicate the cut-off point that relates to the change in the slope of the specific conductivity with respect to the stoichiometry of the complex.

Figure 3 shows the dependence of the specific conductivity against the [Cu^2+^]/[Na_4_BRA] ratio.

**Figure 4:**
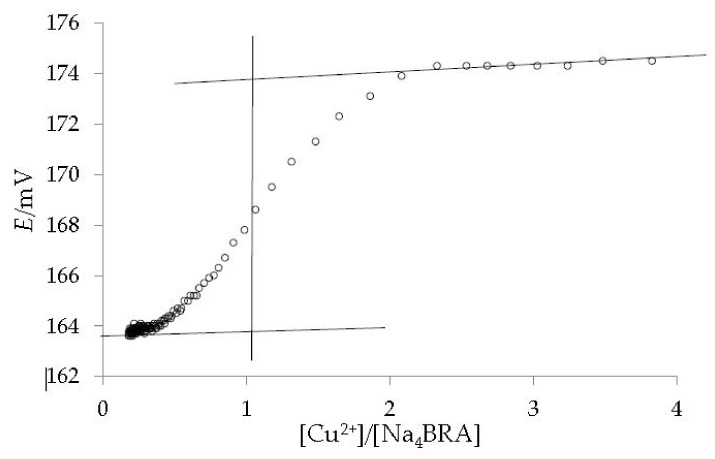
Potentiometric titration of Cu^2+^ with Na_4_BRA, using a copper selective electrode (Cu-ISE). The lines are shown as a visual aid to identify the 1:1 stoichiometry of the complex.

As it can be ascertained, a slight change in slope is observed for a [Cu^2+^]/[Na_4_BRA] ratio of approximately 1.2, which could suggest the presence of a complex of 1:1 stoichiometry. Both the presence of the complex and its stoichiometry were confirmed by potentiometric titration with a copper selective electrode (Cu-ISE). The dependence between the potential and the [Cu^2+^]/[Na_4_BRA] ratio is shown in Figure 4.

A value of Log *β*_ƒ_ = 5.38, at 0.01 M ionic strength was determined for the stability constant of the [BRA-Cu]^2−^ complex using the Hyperquad software [44,45]. In this sense, higher values are reported in the literature for the stability constants of complexes formed by various metals with p-sulfonatocalixarenes [46].

### 3.2. Complexation of Na_4_BRA with Pb^2+^

**Figure 5:**
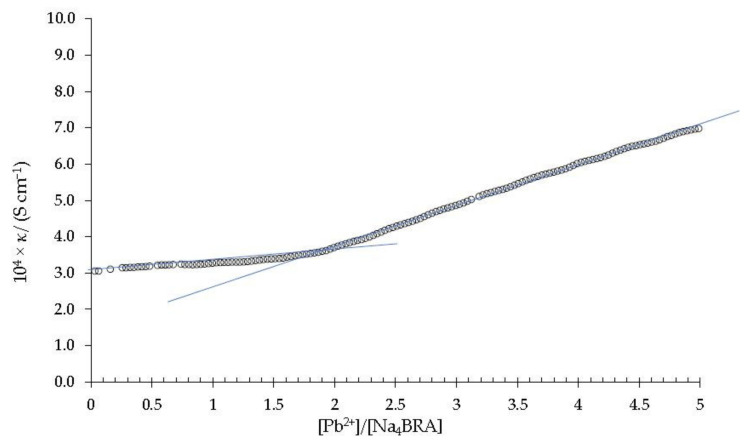
Specific conductivity *versus* the [Pb^2+^]/[Na_4_BRA] ratio. The solid blue lines are shown as a visual aid to indicate the cut-off point that relates the change in slope of the specific conductivity with respect to the stoichiometry of the complex.

Figure 5 shows the dependence of the specific conductivity against the [Pb^2+^]/[Na_4_BRA] ratio.

As it can be seen, an abrupt change in slope appears at a value of 1.9 in the [Pb^2+^]/[Na_4_BRA] ratio, approximately. According to Ashram [47] and Jalali and colbs. the abrupt changes in the slopes of these specific conductivity plots are related to the formation of stable complexes [41]. Therefore, this suggests the presence of a strong and stable complex between Na_4_BRA and Pb^2+^, BRA-Pb, of 1:2 stoichiometry. On the other hand, since this complex precipitates approximately three hours after titration as a fine pink powder [25], it was isolated, purified and analyzed by atomic absorption as indicated in the previous section. The lead content determined in the sample was 25%, which corresponds to a 1:2 stoichiometry for the BRA-Pb complex. This result agrees perfectly with that obtained by conductometric titration and can be explained as the result of the electrostatic interactions between the four sulfonate groups of BRA^4−^ and two Pb^+2^ cations.

### 3.3. Complexation of Na_4_BRA with either Cd^2+^ or Hg^2+^

**Figure 6:**
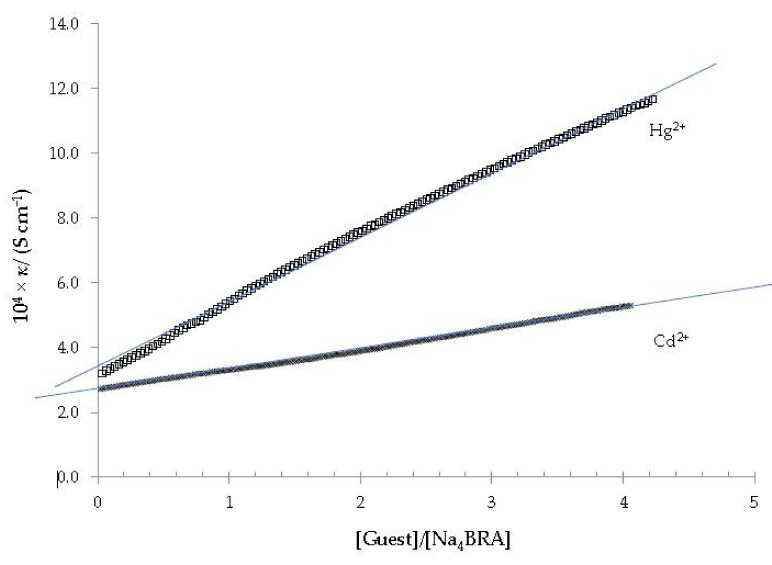
Specific conductivity *versus* the [Hg^2+^]/[Na_4_BRA] and [Cd^2+^]/[Na_4_BRA] ratios. The solid blue lines are displayed as a visual aid to see that there is no appreciable change in the slope of the plot.

Figure 6 shows the dependence between the specific conductivity and the [Hg^2+^]/[Na_4_BRA] and [Cd^2+^]/[Na_4_BRA] ratios.

As it can be seen in both cases, the appearance of a change in slope is not observed in these plots, so the formation of Cd^2+^ or Hg^2+^ complexes with Na_4_BRA must be ruled out.

### 3.4. Complexation of Na_4_SRA with Hg^2+^

**Figure 7:**
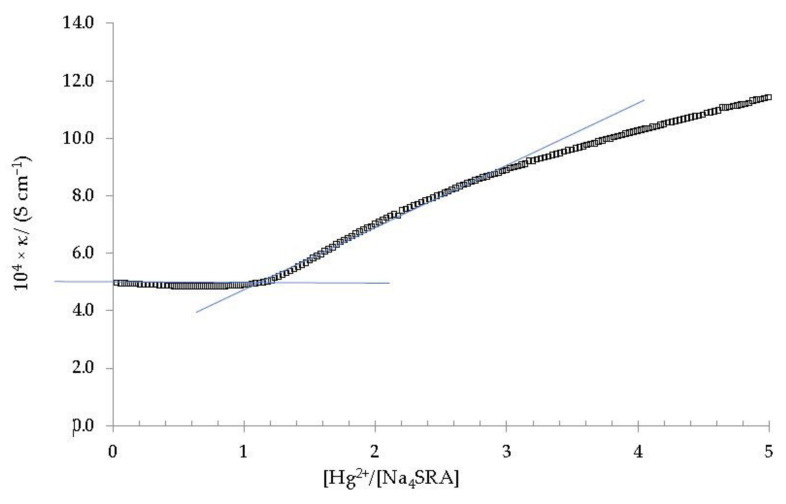
Specific conductivity *versus* the [Hg^2+^]/[Na_4_SRA] ratio. The solid blue lines are shown as a visual aid to indicate the cut-off point that relates the change in slope of the specific conductivity with respect to the stoichiometry of the complex.

Figure 7 shows the dependence of the specific conductivity with the [Hg^2+^]/[Na_4_SRA] ratio.

As it can be seen, there is a significant change in the slope of the plot for a ratio [Hg^2+^]/[Na_4_SRA] equal to 1, which clearly indicates the formation of a stable complex of 1:1 stoichiometry [41,47]. As in the case of [BRA-Pb], this [SRA-Hg] complex precipitates approximately three hours after the end of the titration, appearing as a fine pale-yellow powder [25]. This precipitate was isolated, purified and analysed by atomic absorption to confirm its 1:1 stoichiometry.

The affinity for mercury of sulfur-containing compounds has already been reported by several authors [48,49]. In the case of Na_4_SRA, this can be explained as a consequence of the interactions between the Hg^2+^ ions and the sulfur donor atoms present at the lower rim of the Na_4_SRA.

### 3.5. Complexation of Na_4_SRA with either Cu^2+^, Pb^2+^, or Cd^2+^

**Figure 8:**
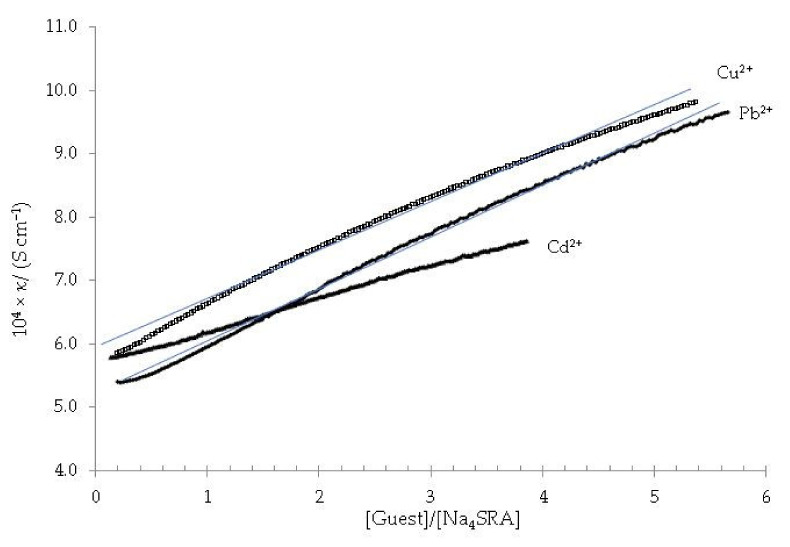
Specific conductivity *versus* the [guest]/[Na_4_SRA] ratio; being the guest (Δ) Pb^2+^, (◊) Cd^2+^, (•) Cu^2+^. The solid blue lines are displayed as a visual aid to see that there is no appreciable change in the slope of the plots.

Figure 8 shows the dependence of specific conductivity against the [guest]/[Na_4_SRA] ratio.

As it is ascertained, in no case a change in the slope is observed, so it must be deduced that none of the Na_4_SRA-Pb^2+^, Na_4_SRA-Cu^2+^ and Na_4_SRA-Cd^2+^ systems show complexation.

Table 3 summarizes what has been seen so far, indicating which guest metals form complexes with the hosts Na_4_BRA and Na_4_SRA.

According to the above table, Na_4_SRA selectively complexes Hg^2+^, while Na_4_BRA complexes Cu^2+^ and Pb^2+^. The fact that Na_4_BRA complexes two guests and Na_4_SRA only one can be explained on the basis of the greater conformational stability of Na_4_BRA compared to Na_4_SRA, caused by Van der Waals-type interactions between the lower edge chains of Na_4_BRA, whereas in the case of Na_4_SRA, conformational mobility can prevent the entry or facilitate the exit of the guest through the upper edge of the host. Additionally, due to the presence of sulfur at the lower edge of Na_4_SRA, this can form bonds with hydrogen from water, which could prevent the inclusion of some guests within the host. In the case of the [SRA-Hg]^2+^ complex, this situation does not occur since mercury can interact with the sulfur donor atoms of Na_4_SRA.

## 4. Conclusions

The complexing properties of Na_4_BRA and Na_4_SRA were studied by conductometry, and the positive results were confirmed by other techniques such as ion-selective potentiometry (ISE) and atomic absorption. The results indicate that Na_4_BRA is able to complex Cu^2+^ in a 1:1 ratio and Pb^2+^ in a 1:2 ratio. Regarding the complex formed by Na_4_SRA with Hg^2+^, the ratio found was 1:1. In this latter case, the results suggest that the interaction between the Hg^2+^ ions and the sulfur atoms present at the lower edge of the SRA^4−^ is greater than that established between these latter atoms and the hydrogens of the solvent (an interaction that would prevent the entry of the guest into the cavity of the SRA^4−^), which favors the entry of Hg^2+^ and means that the Na_4_SRA are selective for these ions.

## Figures and Tables

**Table 1:** Techniques used to study the complexation of heavy metals with Na_4_BRA and Na_4_SRA.

Host	Cu^2+^	Pb^2+^	Cd^2+^	Hg^2+^
Na_4_BRA	Conductometry Ion-selective potentiometry (ISE)	Conductometry Atomic absorption	·Conductometry	Conductometry
Na_4_SRA	Conductometry	Conductometry	·Conductometry	Conductometry Atomic absorption

**Table 2:** Heavy metal salts used for the complexation study with Na_4_BRA and Na_4_SRA.

Substance	Source	Purity
Cu(ClO_4_)_2_·6H_2_O	Alfa Aesar	>98%
Pb(ClO_4_)_2_·3H_2_O	Alfa Aesar	97% min.
Cd(ClO_4_)_2_·6H_2_O	Alfa Aesar	>99%
Hg(ClO_4_)_2_·3H_2_O	Alfa Aesar	>99%

**Table 3:** Stoichiometry of the complexes formed with the hosts Na_4_BRA and Na_4_SRA.

Host/Guest	Cu^2+^	Pb^2+^	Cd^2+^	Hg^2+^
Na_4_BRA	1:1	1:2	-	-
Na_4_SRA	-	-	-	1:1

## Data Availability

Not applicable.
